# Supplementary figures and images for: Improved whole-mount immunofluorescence protocol for consistent and robust labeling of adult Drosophila melanogaster adipose tissue
Source: Biol Open. 2024 Aug 1;13(8):bio060491. doi: 10.1242/bio.060491 (PMC11317099; doi:10.1242/bio.060491)

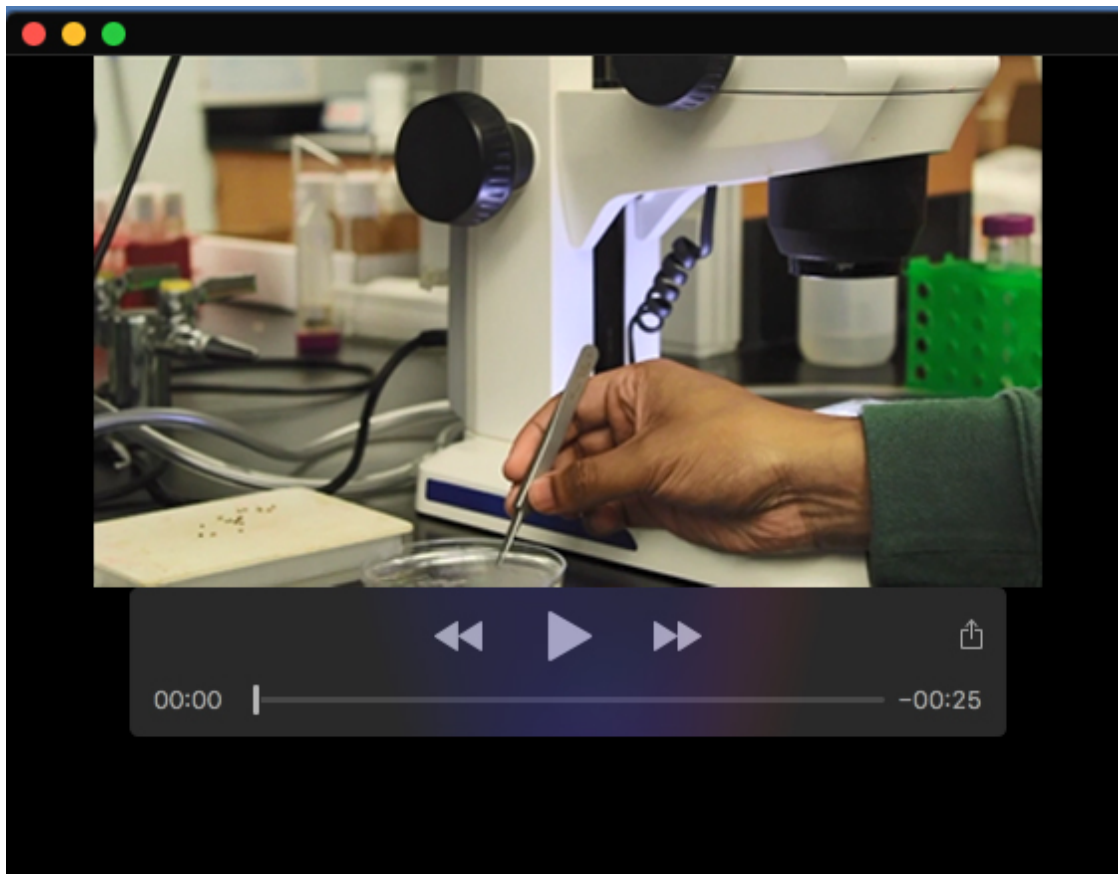

**Movie 1.**

Supplement: Supplementary information [file biolopen-13-060491-s1.pdf]
